# Application of Deep Convolutional Neural Network for Automatic Detection of Digital Optical Fiber Repeater

**DOI:** 10.3390/s22197257

**Published:** 2022-09-24

**Authors:** Xingkang Tian, Fan Wu, Cong Zhang, Wenhao Fan, Yuanan Liu

**Affiliations:** School of Electronic Engineering, Beĳing University of Posts and Telecommunications, Beijing 100088, China

**Keywords:** digital optical fiber repeater, automatic detection, measurement report data, deep learning

## Abstract

The digital optical fiber repeater (DOFR) is an important infrastructure in the LTE networks, which solve the problem of poor regional signal quality. Various types of conventional measurement data from the LTE network cannot indicate whether a working DOFR is present in the cell. Currently, the detection of DOFRs relies solely on maintenance engineers for field detection. Manual detection methods are not timely or efficient, because of the large number and wide geographical distribution of DOFRs. Implementing automatic detection of DOFR can reduce the maintenance cost for mobile network operators. We treat the DOFR detection problem as a classification problem and employ a deep convolutional neural network (DCNN) to tackle it. The measurement report (MR) we used in this paper are tabular data, which is not an ideal input for DCNN. We propose a novel MR representation method that takes the overall MR data of a cell as a sample rather than a single record in the table, and represents the MR data as a pseudo-image matrix (PIM). The PIM will be used as the input for training DCNN, and the trained DCNN will be used to perform DOFR detection tasks. We conducted a series of experiments on real MR data, and the classification accuracy can achieve 93%. The proposed AI-based method can effectively detect the DOFR in a cell.

## 1. Introduction

With the continuous expansion of mobile communication users and the continuous improvement of mobile wireless networks, the number of infrastructure in mobile wireless networks is increasing. The complexity of mobile networks makes detecting and maintaining infrastructure difficult. Achieving efficient detection and rapid maintenance of infrastructure has become a problem for operators.

**Figure 1:**
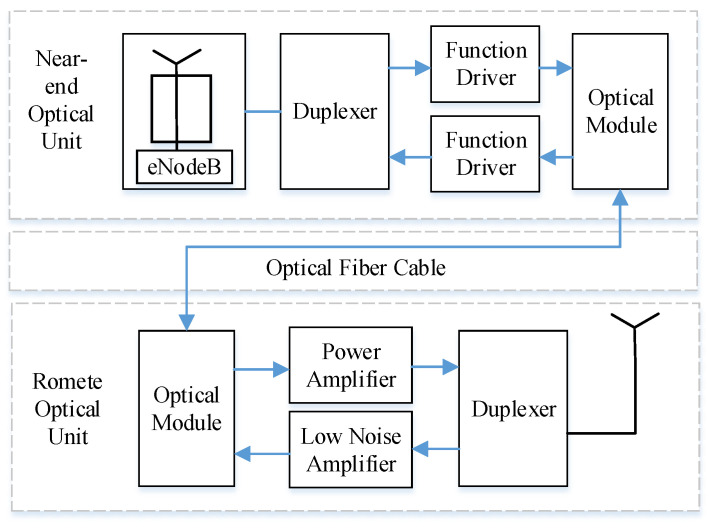
Structural composition of DOFR.

Over the last few decades, consumer groups demand increasingly high requirements for the quality of network usage. Blind areas and weak signal areas still exist in the deployment of the fourth-generation communication network. LTE networks are facing challenges such as providing greater system capacity and wider cell coverage in a cost-effective manner. The installation of additional base stations is complex and expensive, and the corresponding rate of return is low. In contrast, using a digital optical fiber repeater (DOFR) is flexible, simple, and cheap, thereby providing an economical and efficient solution for the network coverage by network operators. The DOFR is a kind of relay device that can enhance the wireless signal, thereby achieving signal enhancement in areas with weak signal coverage. The structure of the DOFR mainly includes the near-end optical unit, the remote optical unit, and the optical fiber cable, as shown in Figure 1.

DOFR is widely deployed to solve the problem of poor signal quality, and has become an important infrastructure in LTE networks. The failure of DOFR will lead to the evident decline of communication quality. Fiber repeaters are deployed by operators, but it is difficult for operators to grasp the operation of all equipment. Because most optical fiber repeaters are old equipment, they cannot be included in the management of the network management system. When the operating status of the equipment changes, the maintenance engineer cannot know it in time. At present, the detection and maintenance of the DOFR are carried out by engineers, which consumes a large amount of manpower. DOFR is an essential infrastructure in LTE networks, which is the reason why automatic detection of DOFR is the key to ensuring automatic maintenance of LTE networks. In addition, it is very important to find out whether DOFRs exist in the cell for making the network optimization scheme.

At present, 5G network construction has entered the stage of large-scale deployment, and mobile network operators have begun to gradually reduce the large-scale construction of 4G networks. 5G network construction has become the current focus of mobile network operators. However, in order for users to transition seamlessly, 4G and 5G will coexist for a long time. The problem of detection of DOFR will not go away with 5G. On the contrary, with the enhancement of the overall operation and maintenance requirements of the mobile network, it will become an urgent problem to be solved.

The large amount of data available on mobile wireless networks has attracted increasing attention. In the research of mobile network big data [1,2,3,4], researchers believe that mobile big data contains a wealth of information to be explored, which brings new opportunities and challenges to mobile wireless networks. Machine learning and deep learning are also increasingly used in the field of mobile wireless networks. The intersection of deep learning and wireless networks is described in Ref. [5]. The mobile wireless network contains various data such as call detail records (CDRs) and measurement report (MR), which can provide data support for automatic operation and maintenance.

In this paper, we conducted relevant research on the detection of DOFR in LTE network on the basis of MR data. The main contributions of this paper can be summarized as follows:

1. We propose a method to represent the MR data of a cell, that realizes the conversion of MR data from tabular to unstructured, and propose the corresponding DCNN model;
2. We use active learning to preferentially select unannotated cells with higher values for annotating to reduce the cost of annotation;
3. We conduct experiments using real MR data, and the detection accuracy of the DOFR reaches 93%, thereby confirming the effectiveness of the proposed method.

## 2. Related Work

The maintenance and optimization of wireless mobile networks require a large number of professionals. The use of automatic methods to reduce labor costs has become a hot topic for researchers. Artificial intelligence (AI)-driven wireless networks are expected to reduce costs and improve user performance from network design to infrastructure management. Empowering future networks with machine learning will enable a shift from incident-driven operations to data-driven operations.

According to industry estimates [6], mobile cellular network operators spend approximately one-fourth of their total revenue on managing and maintaining network resources. Related research [7,8] indicated that the use of big data analysis methods can improve the performance of mobile cellular networks and maximize the revenue of operators. AI provides support for mobile wireless networks, and [9] outlined the integration of AI functions into mobile cellular networks. Ref. [10] pointed out that the use of machine learning technology in mobile wireless networks is expected to reduce operating expenses and improve user experience, and envisaged the use of advanced data analysis and machine learning in wireless networks.

Self-organizing network (SON) technology is considered by Ref. [11] as an effective way to manage and maintain complex networks, improve overall network performance, and reduce operating costs. Ref. [12] used an unsupervised method to implement an SON platform for diagnosing cell faults, which achieved high-precision cell fault diagnosis.

Ref. [13] pointed out that a large part of the maintenance and management costs of mobile cellular networks are used to solve system damage or reduce cellular service failures. Timely and automatic diagnosis of the cause of the failure is critical to maintaining a network. Ref. [14] proposed a method using Bayesian networks to diagnose faults. In Ref. [15], the “if-then” rule was used for automatic troubleshooting of wireless access networks. Ref. [16] proposed a method of using Bayesian network for automatic diagnosis in the universal mobile telecommunication system network. To reduce the participation of human experts, Ref. [13] proposed an AI-based fault diagnosis solution.

In addition to detecting failures that have occurred, network performance prediction allows operators to understand future network conditions and take measures before failures occur. Ref. [17] discussed the application of machine learning techniques for performance prediction problems in wireless networks, and [18] used machine learning to predict uplink power.

In the current study, MR data is widely used for network optimization and maintenance because it reflects the channel conditions. Ref. [19] proposed an interference management algorithm by analyzing MR data. Ref. [20] proposed an LTE network quality analysis method that uses the XGBoost classification algorithm for MR data to quickly diagnose the cause of poor network quality.

Automatic detection of infrastructure is the key to automatic operation and maintenance of an LTE network. This article focuses on the DOFR detection and carries out related research and experiments based on the MR data.

## 3. Deep Learning Based DOFR Detection Using MR

When the status of the DOFR changes in a cell, the MR data that reflect the channel conditions changes accordingly. The MR data obtained from different types of cells have different characteristics. Therefore, we can use the MR data for DOFR detection. We treat the DOFR detection problem as a classification problem and employ a DCNN to tackle it. The original MR data are not suitable for DCNN, thus, we propose a representation method for MR data, which generates unstructured PIMs based on tabular MR data.

### 3.1. Proposed Representation Method for MR Data

DCNN has achieved high performance in computer vision, speech recognition, traffic detection, and other fields, and its application range is gradually expanding. Tabular MR data can neither accurately label the category of a single record, nor is it an ideal input for the DCNN. Therefore, we propose a representation method that consists of three steps of dividing, clustering, and sampling, after which a PIM is generated from the MR data. In the following, we will specifically discuss why and how the table data for each cell should be represented as a PIM.

#### 3.1.1. Advantages of PIM Representation

The MR data are tabular data that contain dozens of features/fields. For the classification task of tabular data, we usually use k-nearest neighbors (k-NN), support vector machine (SVM), random forest (RF), and other methods for supervised learning. MR data does not have record-level annotations, because the operator can only annotate whether a working DOFR is present in the cell, that is, the operator provides cell-level annotations. For the cell without DOFR, we can classify all MR records as not using DOFR, but for a cell that contains DOFRs, the terminal may directly communicate with E-UTRAN NodeB (eNodeB) without using DOFRs. The MR records of the cells containing DOFRs cannot be annotated.

Given the lack of fine-grained record-level annotations for learning, we use coarse-grained cell-level annotations for supervised learning on the basis of the idea of weak supervision [21]. The method of weak supervision is mainly applied in the field of computer vision, where image-level annotations are easier to obtain than pixel-level annotations. For the cell-level label, although we cannot accurately determine which record uses the DOFR, the overall data of a cell with the DOFR include the key records of the use of the DOFR. Representing the cell MR data into a PIM does not lose key information and is easier to annotate, which solves the problem of the inapplicability of fine-grained labels.

#### 3.1.2. Elements of PIM

The MR data contain more than 30 features. On the basis of our analysis, we believe that the four features of TA, RSRP, RSRQ, and PHR are the most important. These features are explained in Table 1.

**Figure 2:**
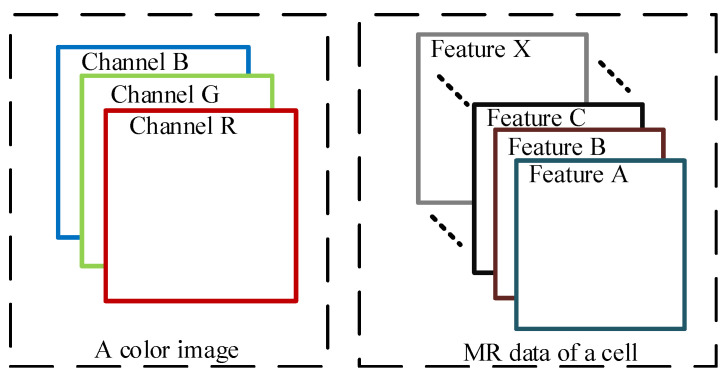
Structural similarity between color image and PIM.

Tabular data are composed of records, and structured data such as images are composed of pixels. Inspired by the way an image is composed, we convert the records into sampling points, and a PIM can be formed by the aggregation of sampling points. A color image is composed of many pixels, and each pixel can be divided into three RGB channels. Each pixel has three attribute values, which can also be considered the features of the pixel. As shown in Figure 2, in the same way, we treat each feature of MR as a channel of the PIM.

The number of MR records in different cells can vary from several thousand to tens of thousands. Image size transformation is a reasonable preprocessing method, but changing the size of the PIM will destroy the original structure of the PIM. To ensure that the size of the PIM is fixed, we avoid the influence of the number of records by sampling according to uniform rules. For the feature dimensions of the PIM, we will discuss in detail in Section 3.4.

#### 3.1.3. Sampling Rules of PIM

We set specific sampling rules for generating PIM. For each record in the PIM, the sampling requirements must be met before being selected.

Orthogonal frequency division multiplexing (OFDM) is a key technology for LTE networks. In order to eliminate the interference caused by the different transmission delays between the terminals and ensure the orthogonality of the uplink, the terminal receives the time advance (TA) command from the network side and adjusts the transmission of control information (including PUCCH/PUSCH/SRS, etc.). Therefore, in a cell where no DOFR is present, combined with transmission time and transmission speed, TA can be converted into transmission distance.

In a cell with DOFRs, a proportional relationship may not exist between the TA and the distance between the terminal and the eNodeB. The terminal in the cell with DOFRs communicates with eNodeB in two ways: 1. The terminal communicates directly with the eNodeB; 2. The terminal communicates through the DOFR. Therefore, for records with the same TA, the TA has two composition modes, as expressed by (1) and (2).

(1) $$TA=2\times delay_{\mathrm{user}\to \mathrm{eNodeB}}$$

(2) $$\begin{array}{ccc} TA & = & 2\times (delay_{\mathrm{user}\to \mathrm{DOFR}}+delay_{\mathrm{process}}\\ & & +delay_{\mathrm{DOFR}\to \mathrm{eNodeB}}) \end{array}$$

On the basis of the differences in the composition of TA between the two types of cells, we choose TA as the primary component of PIM’s location index. In accordance with the composition of the TA, the records of the cell containing DOFR can be divided into two categories: the records that pass through the DOFR and the records that do not pass through DOFR. To better represent the PIM generated by different types of cells and to fully represent whether the cells have DOFRs or not, we cluster the records with the same TA. The size of the PIM needs to be fixed, so the number of clusters should be preset. Considering that K-means can specify the number of clusters and the algorithm converges quickly, we use K-means as the clustering method. The Euclidean distance of features is used as the distance measure for K-means.

**Figure 3:**
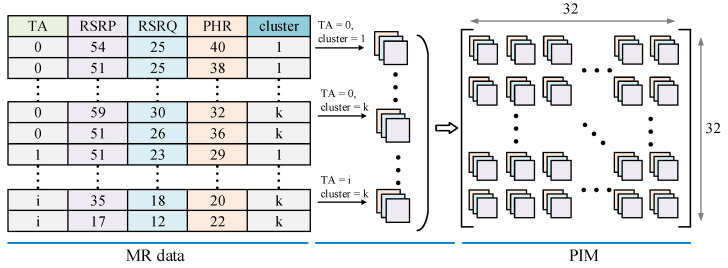
The sampling process of PIM.

We eventually extract elements from each cluster to generate a PIM, as shown in Figure 3.

#### 3.1.4. Generation Process of PIM

In this section, we summarize the process of generating PIM from MR data.

Based on the reasonable range of TA from 0 to 160, we set the plane size of the pseudo-image matrix to 32×32, which means that up to 1024 records are extracted. If no records in a cell meet the TA = i, then PIM is filled with a zero vector. If the number of clusters is 2–6, then the maximum TA varies between 512 and 170. Thus, this range still covers the normal TA.

TA is used as a key position index, and the number of clusters k for the records of the same TA are used to perform clustering. If only a few records satisfy TA, then these records are not of analytical value, and are thus considered unnecessary records. From the k clusters, records are sequentially extracted as the constituent elements of the PIM. We use random sampling during the sampling process. In theory, records with the same TA can be divided into two categories. However, the actual measurement report becomes more complicated because of the environmental factors involved. To ensure that key information is not lost, we set k ⩾ 2.

A cell’s MR data containing m records is expressed as a set $T=\{R_{1},R2,\ldots ,R_{m}\}$, and every record containing n features is expressed as $R_{i}=\left\{R_{i1},R_{i2},\ldots ,R_{in}\right\}$. The process of converting all sampling records into PIM is expressed as *Conversion*. The representation process is shown in Algorithm 1.
**Algorithm 1:** Representation method for MR data.
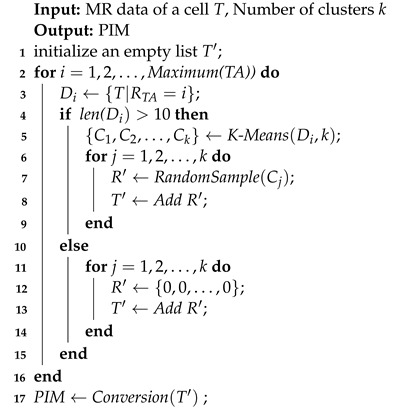


### 3.2. Proposed DCNN Structure

As an important convolutional network model, the ResNet network model has achieved excellent performance in image recognition [22], speech recognition [23], fault diagnosis [24], and other problems. We proposed our DCNN based on the ResNet network structure.

**Figure 4:**
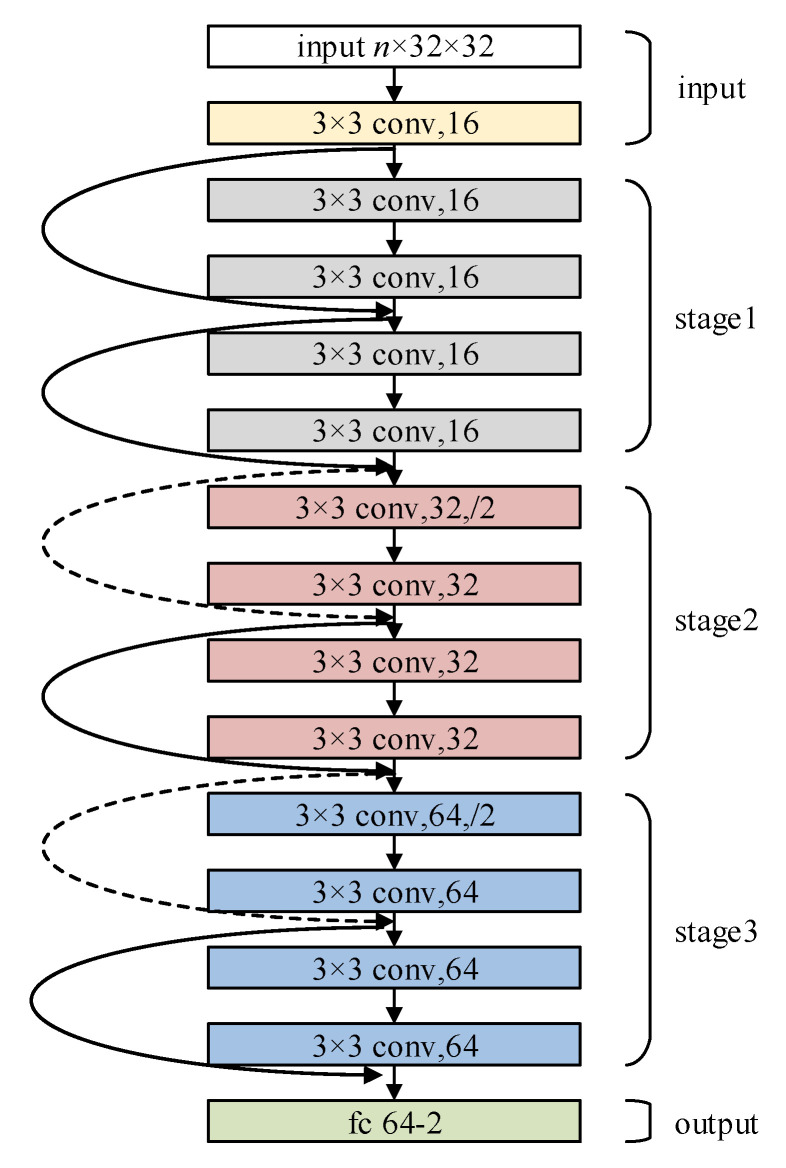
The architecture of the proposed DCNN.

The size of the PIM is n×32×32 (*n*: the number of selected features), and the size is small, thus we built a DCNN based on BasicBlock. The structure of our proposed DCNN is shown in Figure 4.

The overall network structure consists of three parts: the input part, the output part, and the middle convolution part. The middle convolution part includes three stages: stage1, stage2, and stage3. Each stage performs downsampling through a convolutional layer with a step size of 2. Downsampling is completed in the first convolution of each stage only, and and only one occurs in each stage.

After the PIM enters the network, it first passes through the input part: conv, bn, and ReLU. Then it enters the middle convolution part: stage1, stage2, and stage3. Finally, the data pass through the average pooling, fully connected layer, and softmax.

**Figure 5:**
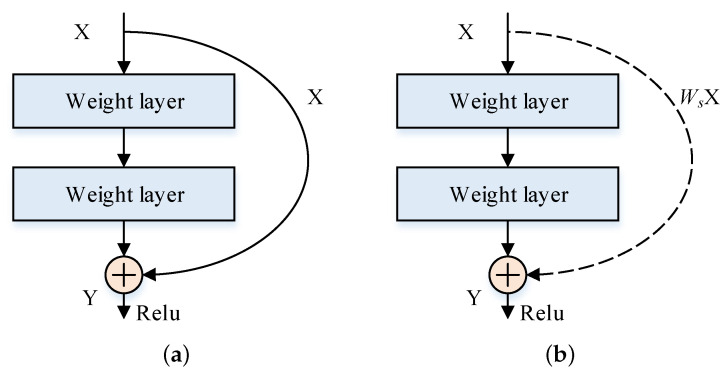
Two modes of shortcut connection. (**a**) Input and output channels are same. (**b**) Input and output channels are different.

The shortcut connection in Figure 4 has two modes. The connection with the same channels is denoted by a solid line in Figure 5a and its calculation method is Y=f(X)+X, The connection with different channels is denoted by a dashed line in Figure 5b and its calculation method is $Y=f\left(X\right)+W_{s}X$.

### 3.3. Preparation for Learning

The number of cells with clear labels that can be used is only 333, including 96 cells with DOFRs and 237 cells without DOFRs. If each cell generates a sample, then the number of PIM is only 333.

Although our DCNN structure is not complicated, hundreds of samples are still insufficient for deep neural network training. To make the model training effective, we propose a method to expand the PIM data set based on the sampling method. The proposed representation method generates PIM by sampling. Even if the MR data of a cell are sampled to form PIM multiple times, the probability of the same PIM is very low. Therefore, we will generate multiple PIMs for the same cell as a data enhancement method to expand our training samples.

In the process of generating the PIM data set, we repeatedly sampled each cell multiple times to increase the number of samples. The number ratio of the two types of cells is not balanced, so we set different sampling numbers for the two types of cells to dynamically adjust the proportion of the PIMs. The proportions of the two types of samples in the data set are ensured to be roughly equal, as expressed in the following formula.

(3) $$N_{P}\times D_{P}\approx N_{N}\times D_{N}$$

where $N_{P}$ is the number of cells with DOFR, $N_{N}$ is the number of cells without DOFR, $D_{P}$ is the number of PIM for the cell with DOFR, and $D_{N}$ is the number of PIMs for the cell without DOFR.

### 3.4. Selecting the Best Parameters of the Representation Method

The proposed representation algorithm has two important parameters: selected features and number of clusters. These two parameters will affect the representation performance of the generated PIM, so we conducted experiments to determine the best parameters of the representation method.

**Figure 6:**
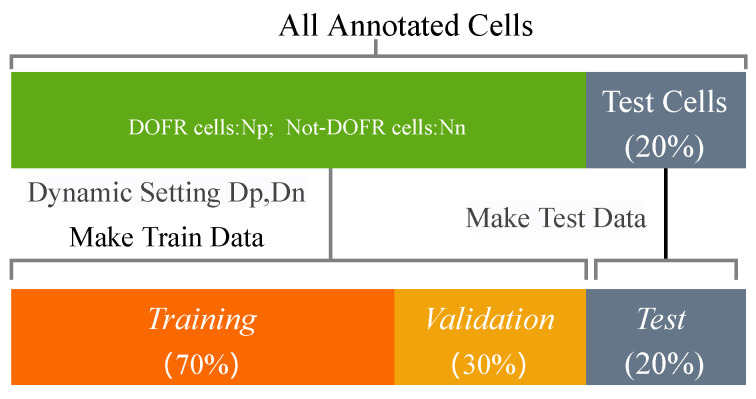
The apportion of cells used for training and testing.

We use different parameter combinations to generate data sets as the input of the DCNN network for experiments. The number of labeled cells is small, thus, we use a five-fold cross-validation method to evaluate the results of different parameter combinations. We separated our total annotation cells almost equally into five segments. $\frac{4}{5}$ cells are used in the training of DCNN while the remainder ($\frac{1}{5}$) cells are used to validate the performance of our proposed method. For the training cells, we dynamically adjust the number of generated samples according to the ratio of the two types of cells to ensure a balanced sample ratio, that is, $N_{P}\times D_{P}\approx N_{N}\times D_{N}$. Figure 6 shows the apportioning of the annotation cells for training and testing. For the test cells, each cell generates five PIMs, and the final result is judged based on the five results.

Because we use TA as a position index in our generation process, the information about TA is already contained in the matrix structure and TA is not used as a feature. We tried all combinations of features separately. In the case of each feature combination, we analyzed the influence of the different cluster numbers. Three evaluation metrics were used: accuracy (A), precision (P), and recall (R). Accuracy was used to evaluate the overall performance of a classifier. Precision and recall were used to evaluate the performance of every class of cells.

(4) $$A=\frac{TP+TN}{TP+FP+FN+TN}$$

(5) $$P=\frac{TP}{TP+FP}$$

(6) $$R=\frac{TP}{TP+FN}$$

where TP is the number of instances that are correctly classified as DOFR cell, TN is the number of instances that are correctly classified as Not-DOFR cell, FP is the number of instances that are incorrectly classified as DOFR cell, and FN is the number of instances that are incorrectly classified as Not-DOFR cell.

**Figure 7:**
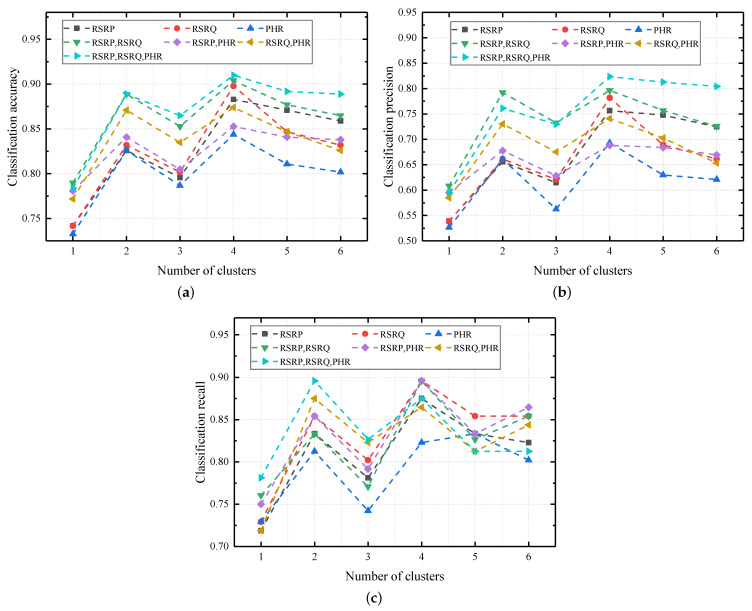
Performance comparison of the feature combinations. (**a**) Classification accuracy. (**b**) Classification precision. (**c**) Classification recall.

The experimental results are shown in Figure 7. When the matrix dimension is 1, feature RSRP and feature RSRQ have better performance and feature PHR has the worst performance. A single feature PHR has poor performance, but when combined with other features, it can achieve performance improvements.

When the number of clusters is set to 4, most feature combinations achieve the best performance. When the number of clusters is 1, no clustering is performed, and the records that meet the TA conditions are randomly selected. Key records may be ignored because of the randomness of the extraction. Therefore, for all feature combinations, when the number of clusters is 1, the performance is the lowest.

**Figure 8:**
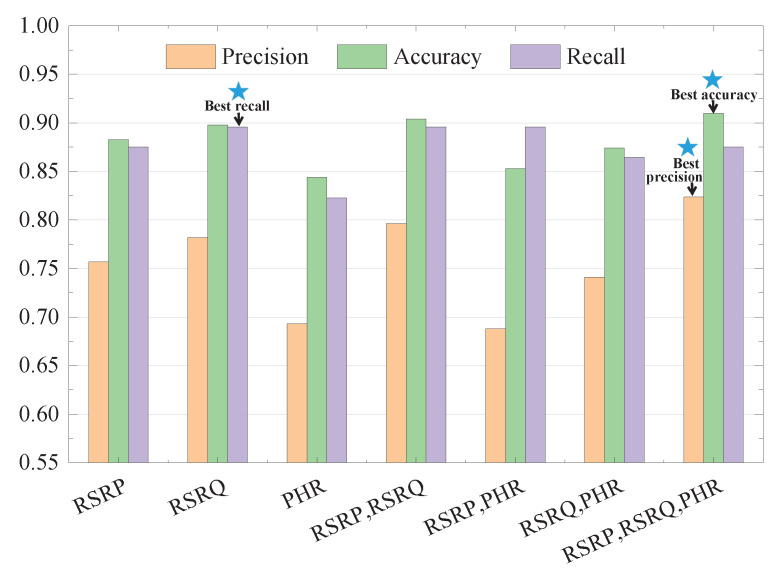
Performance comparison between feature combinations when the number of clusters is 4.

As Figure 8 shows, when the number of clusters is set to 4, the RSRP-RSRQ-PHR has the best precision and accuracy among all feature combinations. The relevant experimental results are recorded in Table 2. So, we choose RSRP-RSRQ-PHR and set the number of clusters to 4.

### 3.5. Comparison with Current Methods

The operator’s current method of analyzing MR data mainly relies on expert experience. According to experience, the operation and maintenance engineer believes that the cell with multiple peaks in the TA distribution map of the MR data is more likely to have an optical fiber repeater. The main basis for the judgment is that the DOFR will introduce processing delay. Therefore, the TA value of the record passing through the DOFR is usually larger. However, in actual experiments, it is found that the accuracy of this analysis is very low, and its accuracy is less than 70%. We analyze that the reason for its low accuracy is mainly that this method is seriously affected by user distribution, and this method only utilizes a single feature of MR data, ignoring many important records.

Compared with current methods, our proposed method makes full use of various features in MR data, and can explore the potential relationship between different features, which effectively improves the detection accuracy.

## 4. Dataset Expansion and Experimental Results

The performance of the DCNN is not very high, and its detection accuracy can reach only 91% at present because of the small amount of sample data. In the actual operation and maintenance process, the prediction results based on the model have significantly improved the efficiency of engineers’ field work. Each operation and maintenance needs to be carried out on site. Compared with the traditional one-by-one operation and maintenance method, the operation and maintenance based on the model prediction result has achieved a great reduction in cost.

The performance of deep neural networks is highly dependent on the size of the data set. We confirm that the performance of the model will increase even more as the amount of data increases. However, DOFRs are widely distributed, especially in rural areas and remote mountainous areas, where the cell annotation is very slow and costly. Aiming at the high cost of cell annotation, we use active learning to preferentially select unannotated cells with a higher value for annotation so that the model can achieve better performance while minimizing annotation costs.

In this section, we propose an interactive annotation process based on active learning approaches. Experiments were conducted in collaboration with mobile network operators using real MR data in a certain area. At the same time, the performance improvement of DCNN during the iteration process was recorded, and the active learning method was used to obtain a high-performance model with the lowest possible annotation cost. More importantly, it proves that as the data set expands, our method has great potential.

### 4.1. Data Source

In this section, we briefly introduce the source of our experimental data.

The raw measurement report was collected to the Operation and Management Center-Radio (OMC-R) to generate MRO files. What we are using is real MRO data from a specific area. By analyzing the MRO data, we obtained the original MR record. Then, we cleaned the original MR data to eliminate abnormal data. Because the MR data is reported periodically by the terminal, the records with a short interval may be the same, so there is a large amount of repeated data. For cell ID, terminal ID, RSRP, RSRQ, PHR, and TA, these six fields of the same duplicate records are also eliminated.

**Figure 9:**
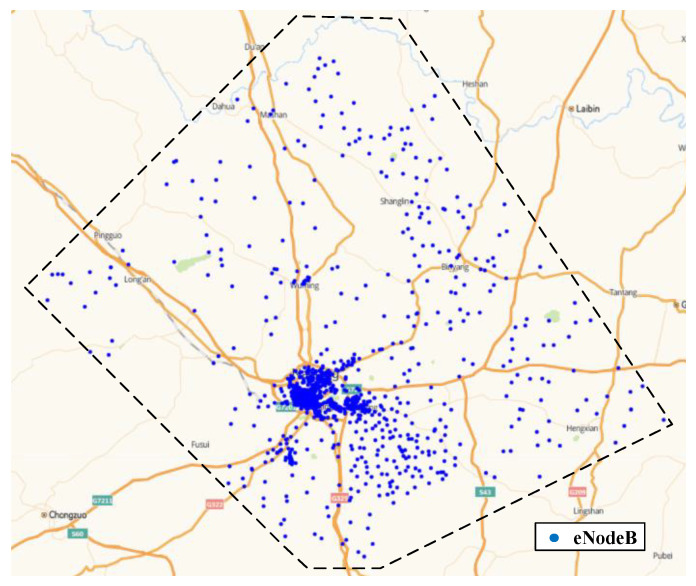
Geographic image of the data source area.

The collection area of experimental data is shown in Figure 9, which contains 1266 eNodeBs. This area contains 9268 cells, of which 333 cells are randomly selected and manually labeled, including 237 cells without DOFRs and 96 cells with DOFRs.

### 4.2. Annotation Process

**Figure 10:**
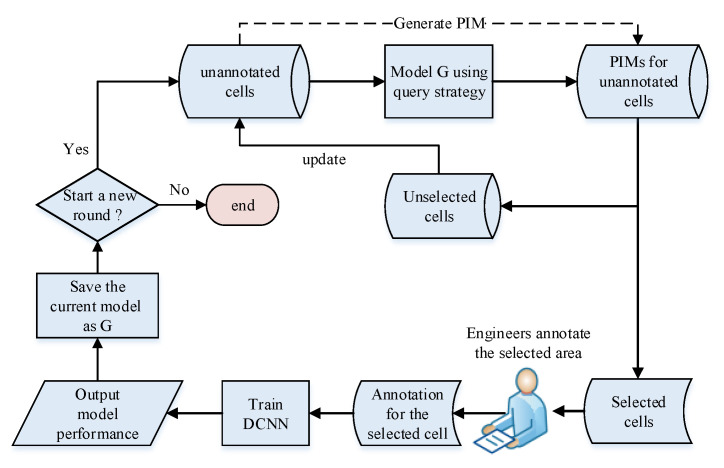
Annotated cell expansion process based on active learning.

To reduce the high cost of cell annotation, we use active learning to preferentially select unannotated cells with a higher value for annotation to avoid the bias and randomness of randomly selecting cells. First, we determine the cells to be annotated from the unannotated cell set according to the select query strategy. Then the maintenance engineer conducts field detection and returns the annotation result. We generate samples of the newly annotated cells and add them to the training data set. Finally, the model is retrained on the updated data set. As the size of the training set continues to increase, the performance of the model continues to improve through the iterative annotation process. The iterative process of active learning is shown in Figure 10.

The select query strategy is directly related to how much the annotation cost can be saved. In the experiment, on the basis of the particularity of the PIM and the deep neural network model used, we used entropy maximization to prioritize the selection of samples with a larger entropy score. The calculation formula for the entropy score (ES) can be expressed as the following equation:

(7) $$ES=-\sum_{i=1}^{2}p_{i}\times log\left(p_{i}\right)$$

where $p_{i}$ represents the probability of class *i*.

To avoid the randomness of a single sample, we generate *N* samples for each unannotated cell, and finally select cells with a larger average entropy score (AES) for priority annotation. The AES is expressed as follows:

(8) $$AES=\frac{1}{N}\sum_{n=1}^{N}ES_{n}$$

The steps to select cells based on AES and update the data set are depicted in Algorithm 2.
**Algorithm 2:** Annotation process.
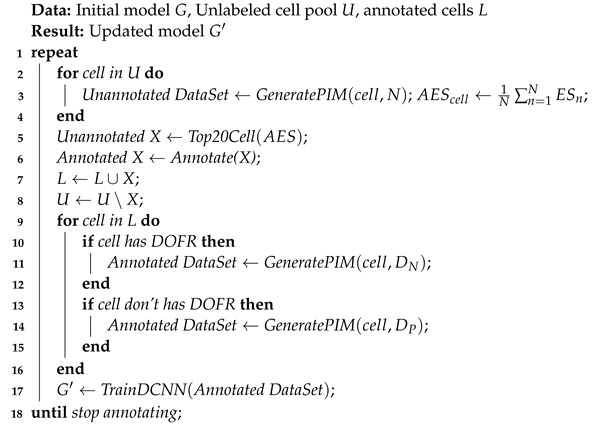


### 4.3. Iteration Result

For unannotated cells, each cell generated five PIMs and calculated the AES of the cells based on these PIMs. In order to reduce the iteration cycle, we selected only the top 20 cells with the largest AES value from the unannotated cell pool for annotation in each iteration. We have performed four iterations so far.

**Figure 11:**
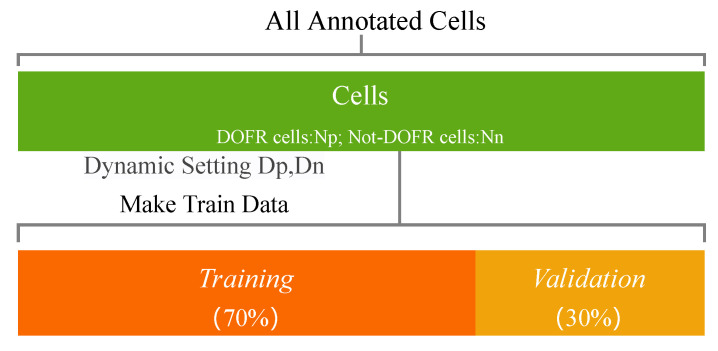
The apportion of all samples for training and validation.

For the performance evaluation of each iteration, we used the same evaluation metrics and data division principles as in Section 3.4. For the prediction of unannotated cells, we used all annotated cells to generate a PIM set, using 70% of the PIM set as the training set and the remaining 30% as the verification set, as shown in Figure 11. The detailed experimental results are recorded in the Table 3.

**Figure 12:**
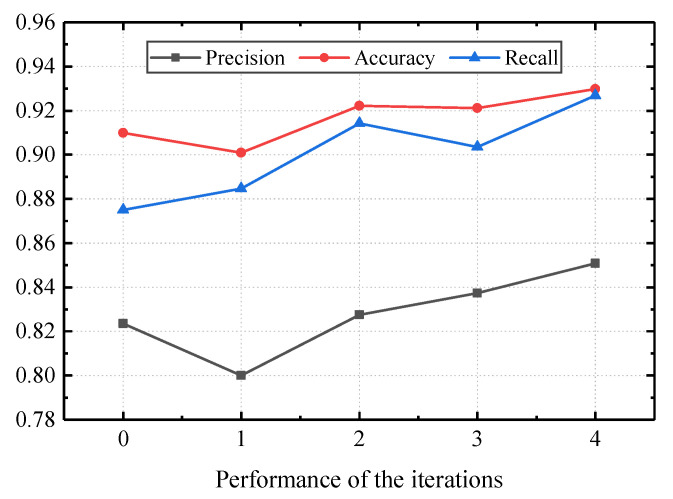
Performance of the iteration experiment.

As shown in Figure 12, as the iterative process progresses, the performance of the model is significantly improved. Although these three broken lines in the figure have slight fluctuations, they eventually show an upward trend. After the fourth round of iteration, the accuracy, precision, and recall of the model reached 93%, 85%, and 93%, respectively, which met the initial expectations of mobile network operators. After the expansion of the annotated cells, the performance of the model has been improved. However, the total amount of data is still not large enough, and further improvement of the model performance requires more cell annotation.

## 5. Conclusions

This work introduced a deep learning approach for automatic detection of DOFR in LTE networks. We proposed a representation method for MR data and designed a DCNN to address the difficulty operators experience in automatically detecting DOFRs. By extracting records, the representation method converts the MR data of different cells into a matrix of the same size. Experiments show the effectiveness of this representation method. In addition, active learning is used to select cells to be annotated, and a network model with higher performance is obtained after multiple rounds of iterative experiments. Experiments show that our model can be used to efficiently solve the problem of automatic detection of DOFR.

## Figures and Tables

**Table 1:** Explanation of important features of MR.

Abbreviation	Explanation
TA	Time Advance
RSRP	Reference Signal Receiving Power
RSRQ	Reference Signal Receiving Quality
PHR	Power Headroom Report

**Table 2:** Experimental results of different feature combinations.

Feature	Precision	Accuracy	Recall
RSRP	0.7568	0.8829	0.8750
RSRQ	0.7818	0.8980	0.8958
PHR	0.6930	0.8438	0.8230
RSRP, RSRQ	0.7963	0.9039	0.8958
RSRP, PHR	0.6880	0.8529	0.8958
RSRQ, PHR	0.7411	0.8739	0.8646
RSRP, RSRQ, PHR	0.8235	0.9099	0.8750

**Table 3:** Experimental results of the iteration.

Iteration	Precision	Accuracy	Recall
0	0.8235	0.9099	0.8750
1	0.8001	0.9008	0.8846
2	0.8276	0.9223	0.9143
3	0.8374	0.9211	0.9035
4	0.8508	0.9298	0.9268

## Data Availability

Not applicable.

## Abbreviations

The following abbreviations are used in this manuscript:

DOFR	Digital Optical Fiber Repeater
DCNN	Deep Convolutional Neural Network
MR	Measurement Report
PIM	Pseudo-Image Matrix
TA	Time Advance
RSRP	Reference Signal Receiving Power
RSRQ	Reference Signal Receiving Quality
PHR	Power Headroom Report
